# Cellular senescence in ischemia/reperfusion injury

**DOI:** 10.1038/s41420-022-01205-z

**Published:** 2022-10-18

**Authors:** Chaojin Chen, Muxu Zheng, Hongbiao Hou, Sijian Fang, Liubing Chen, Jing Yang, Weifeng Yao, Qi Zhang, Ziqing Hei

**Affiliations:** 1 Department of Anesthesiology, The Third Affiliated Hospital of Sun Yat‑sen University, 510630 Guangzhou, Guangdong P. R. China; 2 Cell-gene Therapy Translational Medicine Research Center, The Third Affiliated Hospital of Sun Yat-sen University, 510630 Guangzhou, Guangdong P. R. China; 3 School of Medicine, Sun Yat-sen University, 518107 Shenzhen, Guangdong P. R. China

**Keywords:** Senescence, Ageing, Kidney

## Abstract

Ischemia/reperfusion (IR) injury, a main reason of mortality and morbidity worldwide, occurs in many organs and tissues. As a result of IR injury, senescent cells can accumulate in multiple organs. Increasing evidence shows that cellular senescence is the underlying mechanism that transforms an acute organ injury into a chronic one. Several recent studies suggest senescent cells can be targeted for the prevention or elimination of acute and chronic organ injury induced by IR. In this review, we concisely introduce the underlying mechanism and the pivotal role of premature senescence in the transition from acute to chronic IR injuries. Special focus is laid on recent advances in the mechanisms as well as on the basic and clinical research, targeting cellular senescence in multi-organ IR injuries. Besides, the potential directions in this field are discussed in the end. Together, the recent advances reviewed here will act as a comprehensive overview of the roles of cellular senescence in IR injury, which could be of great significance for the design of related studies, or as a guide for potential therapeutic target.

## Facts

- Ischemia/reperfusion(IR) injury is an inevitable pathological process in many clinical practices, such as transplant surgery, emergency rescue after shock, myocardial infarction and so on. The morbidity and mortality owing to IR injury remain high although many treatments have been developed to prevent this process.
- Cellular senescence is a state of irreversible cell-cycle arrest that could be caused by many stresses, including IR injury, which is called Stress-induced Cellular Senescence(SIPS).
- SIPS plays a key role in IR-induced acute and chronic tissue damage/dysfunction of multiple organs, especially in kidney, heart and brain.

## Open Questions

- Currently, the underlying mechanism of IR-induced acute to chronic organ and tissue damage remains complicated and obscure. Therefore, it is of great significance that further study needs to be carried out.
- Cellular senescence serves as a core mechanism in IR-induced acute to chronic organ and tissue damage. However, the specific mechanism underlying senescence-induced acute and chronic tissue damage remains unclear and needs to be clarified in the future.
- A large number of reports have confirmed that cellular senescence may serve as a therapeutic target to alleviate IR-induced tissue and organ injury. Hence, the development of treatments targeting cellular senescence will have a wide clinical application in IR-induced acute and chronic injury.

## Introduction

Ischemia/reperfusion (IR) injury refers to a condition when the tissues or organs suffer from restricted blood supply, the recovery of blood supply and perfusion do not alleviate ischemic injury, conversely lead to further damage/dysfunction. It is an inevitable pathological process in many clinical practice, such as transplant surgery, emergency rescue after shock, and myocardial infarction. Despite techniques such as thrombolytic therapy, percutaneous coronary angioplasty, and cardiopulmonary bypass have made incredible advancements in reducing tissue ischemia, the morbidity and mortality owing to IR injury after operation still remain high. Extensive studies are focusing on investigating the underline mechanisms of IR injury, involving oxidative stress [1–4], calcium overload [1, 3], mitochondrial dysfunction [3, 5, 6] and excessive inflammation [1, 3, 7, 8]. These multiple signaling pathways are interrelated and interactive, which eventually contribute to different kinds of cell phenotypes due to different environments and extents of damage: apoptosis [7, 9], necroptosis [9–12], necrosis [1, 2, 7], pyroptosis [9, 13, 14], ferroptosis [15–17] and cellular senescence [18]. However, the full picture of the pathophysiology of IR injury is far from complete and further research is needed.

**Fig. 1:**
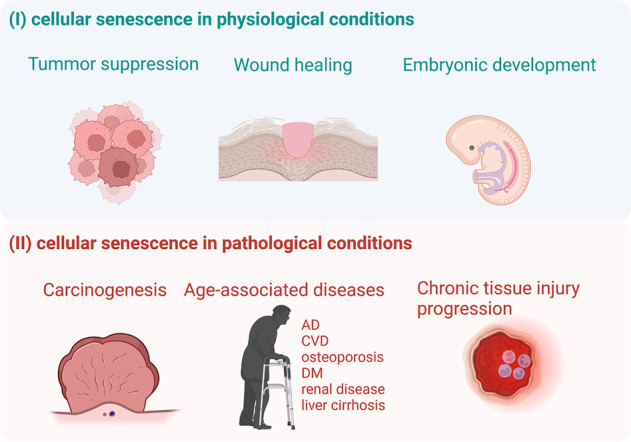
Role of cellular senescence in physiology and pathology conditions. Cellular senescence plays dual roles in different conditions. In physiological conditions, cellular senescence contributes to tumor suppression, wound healing, and embryonic development. Cellular senescence is thought to have evolved as an antitumor mechanism where the senescence-associated secretory phenotype(SASP) recruits immune cells to facilitate senescent cells removal. In embryonic development and wound, cell-cycle arrest is induced in damaged cells and results in their elimination by macrophage. Nevertheless, in pathological conditions, senescent cells may result in carcinogenesis if they exist for a long time without clearance. Cellular senescence can also contribute to different kinds of age-associated diseases (such as Alzheimer’s diseases, cardiovascular diseases, osteoporosis, diabetes, renal disease, and liver cirrhosis) and chronic tissue injury progression.

**Fig. 2:**
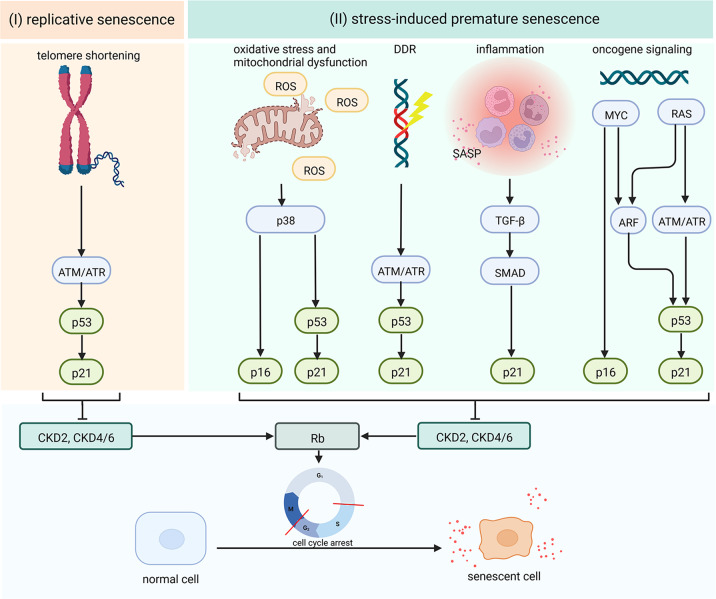
Cellular senescence signaling pathways. The internal mechanism that leads to cellular senescence varies depending on the triggers and context. Several pathways contribute to the activation of cell-cycle inhibitors, inhibition of retinoblastoma protein (RB) phosphorylation, and cell-cycle arrest which is the main manifestation of cellular senescence. The production of various chemokines, inflammatory cytokines, growth factors, and extracellular matrix remodeling factors which are named “senescence-associated secretory phenotype” (SASP) is also another significant manifestation of cellular senescence. Cellular senescence can be divided into replicative senescence and stress-induced premature senescence(SIPS). (I) In replicative senescence, telomere shortening may trigger activation of ataxia telangiectasia mutated (ATM) or ataxia telangiectasia and RAD3-related protein (ATR) kinases, and result in p53 upregulation, and increased p21. (II) In stress-induced premature senescence, mitochondrial dysfunction and oxidative stress may activate the mitogen-activated protein kinase kinase (MKK3 and MKK6) pathway and their downstream effector p38, leading to the upregulation of p16, p53, and p21 level. DNA damage activates a signaling cascade via ATM/ATR kinases, p53 upregulation, and increased p21. In inflammation response, a component of the senescence-associated secretory phenotype (SASP) pathway named transforming growth factor-β (TGF-β), may upregulate p21 level through SMAD complexes. Lastly, oncogenic signaling or loss of tumor suppressors upregulates p16, p53, and p21 levels, mediated by RAS, MYC, and phosphoinositide 3-kinase (PI3K) and their downstream effectors ATM, ATR, and ARF.

Cellular senescence refers to the state of nonreversible cell-cycle arrest that plays dual roles in different conditions [19, 20] (Fig. 1). In IR-induced acute and chronic tissue damage/dysfunction, cellular senescence is of great significance. In general, senescence can be divided into replicative senescence and premature senescence [21](Fig. 2). Different from replicative senescence induced by telomere shortening after repeated cell division, premature senescence refers to the situation that accelerated by numerous stressful stimuli such as oxidative stress, DNA damage, mitochondrial dysfunction, inflammation, activated oncogenes [22, 23], which is also described as stress-induced premature senescence (SIPS). For the most part, cellular senescence is triggered primarily by the activation of p53/p21^CIP1^ and/or p16/pRb pathways and characterized as G1/S (or G2/M) cell-cycle arrest [22, 24–26]. Moreover, cellular senescence can induce the production of various chemokines, inflammatory cytokines, extracellular matrix remodeling factors, and growth factors by “senescence-associated secretory phenotype” (SASP), including IL-1, IL-6, PAI-1, TGF-β, CTGF, CCN2, and MCP-1 [27–32].

In recent years, studies have confirmed that cellular senescence regulates the development and progression of IR-induced acute and chronic diseases in different organs dynamically and reversibly [33]. More and more evidence revealed that genetic and pharmacological clearance of senescent cells could ameliorate IR-induced acute and chronic disease [34–37]. However, cellular senescence induced by IR injury is extremely complex and has not yet been fully elucidated. Here, we focus on the recent advances in the role and mechanism of cellular senescence in IR-induced acute and chronic injury in different organs, and clarify the basic and clinical treatment options aiming at treating IR-induced cellular senescence in diverse organs.

## Recent advances in ischemia/reperfusion-induced senescence machinery

**Fig. 3:**
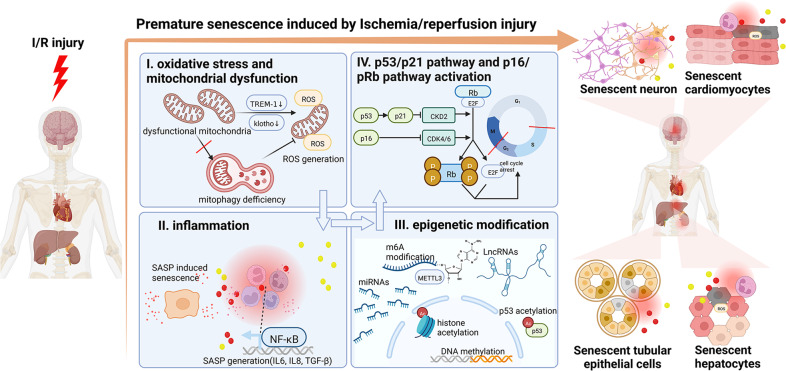
Premature senescence induced by ischemia/reperfusion injury. IR injury first initiates (I) oxidative stress and mitochondrial dysfunction, followed by (II) inflammation and (III) epigenetic modification, finally activates (IV) p53/p21 and p16 senescence pathway and cause cellular senescence. (I) IR injury may damage the function of mitochondria in parenchymal cells such as renal tubular epithelial cells, neurons, cardiomyocytes, and hepatocytes, lead to ROS generation through downregulation of TREM-1 and klotho and can also mediated by mitophagy defficiency. (II) Inflammation response is characterized by infiltration of immune cells such as macrophages, neutrophils, and lymphocytes in the mesenchyme which are recruited by ROS generated by oxidative stress initially. The infiltrating inflammatory cells will release pro-inflammatory factors (also known as SASP if released by senescent cells) such as IL-6 and IL-8. Besides, senescent parenchymal cells can also release SASP to amplify the inflammation response. (III) Multiple kinds of epigenetic modification including m6A modification, DNA methylation, histone, and p53 acetylation, miRNAs and LncRNAs are involved in IR-induced senescence. (IV) p53/p21 pathway and p16 pathway are the final signaling to induce cellular senescence.

Consistent with other premature senescence caused by various stressful stimuli, the mechanism underlying IR-induced cellular senescence is complex, including oxidative stress, mitochondrial dysfunction, mitophagy deficiency, inflammation response, and epigenetic modification, which finally contribute to the activation of p53/p21^CIP1^ and/or p16/pRb senescent pathway (Fig. 3).

### Mitochondrial dysfunction and oxidative stress

IR injury is primarily characterized by mitochondrial dysfunction and burst production of reactive oxygen species (ROS). The excessive production of ROS causes oxidative stress in tissues, leading to cell death and ultimately organ dysfunction [38]. Growing evidence showed that mitochondrial dysfunction and oxidative stress induced by IR might further result in cellular senescence, mediated by p21 and p16 signaling activation [39].

In terms of the internal mechanism, recent studies have shown that mitochondrial autophagy (mitophagy) deficiency after renal IR would alter mitochondrial network and cause the accumulation of dysfunctional mitochondria, which led to excessive ROS-induced senescence [40, 41]. In addition, Miao et al. reported that IR would inhibit Klotho, a widely reported factor associated with anti-senescence, leading to the activation of Wnt1- and Wnt9a-induced mitochondrial injury and cellular senescence in renal [18]. Meanwhile, Tammaro et al. recently indicated that deficiency of the triggering receptor expressed on myeloid cells-1 (TREM-1), an innate immune receptor, would damage mitochondrial metabolism, increase ROS accumulation, drive G2/M arrest and senescence in tubular epithelial cells after renal IR [42]. Moreover, IR can accelerate mitochondrial fission–associated myocardial senescence in mice, following myocardial infarction [43]. These discoveries are consistent with the hypothesis that mitochondrial dysfunction and oxidative stress are involved in IR-induced senescence.

### Inflammation

The increased generation of ROS and mtDNA after IR, which are also named damage-associated molecular patterns(DAMPs), would contribute to neutrophil infiltration and a large amount of pro-inflammatory cytokines release, which plays a crucial role in cellular senescence. Increasing studies have shown a complicated interaction between senescence and inflammation in IR injury [44]. On the one hand, the overactive inflammatory response is one of the major predispositions to SIPS. On the other hand, senescent cells may give rise to senescence of their nearby cells through the SASP, amplifying the inflammatory response that follows [45, 46].

As for the internal mechanism, inflammation is considered a complicated interaction between immune cells and parenchymal cells [47] and marked by infiltration of immune cell in the mesenchyme [48]. In the early phases of IR injury, neutrophils are recruited by DAMPs mainly [49]. These DAMPs, interact with pattern recognition receptors(PRRs) on macrophages and contribute to their activation [50], and thus promote cell-cycle arrest through SASP [51]. Another kind of innate immune cells, Dendritic cells(DCs), which serve as a mediator of the recruitment and activation of effector T cells, promoting interstitial immune response [52]. DCs would also aggravate SASP production in immune cells in cisplatin-induced AKI, which serve as crucial amplifiers of local innate immune responses in AKI [53]. These studies emphasize the importance of inflammation microenvironment in cellular senescence. Meanwhile, Qian Li et al. pointed out that renal sympathetic neurotransmitter NE, acting on the α_2A_-AR of epithelial cells, could promote the crosstalk between inflammation and cellular senescence, contributing to renal fibrosis after IR injury [54]. Weifeng Yao et al. found that aerosol inhalation of a hydrogen-rich solution would attenuate renal macrophage infiltration, the release of pro-inflammatory cytokine, and cellular senescence via TGF-β1 pathway in septic acute kidney injury (AKI) [55]. Besides, inflammation and senescence share a cascade amplification process with each other in cardiac [56] and hepatic IR injury [57]. Recently, Qi et al. found that inhibition of NF-κB pathway would disrupt the reciprocal cycle between inflammation and senescence of TECs [58], and the elimination of senescent myocardiocytes after MI would markedly reduce SASP and induce efferocytosis of macrophage to downregulate inflammation [59]. Putting it all together, further exploring the positive feedback loop between inflammation and cellular senescence might help to alleviate multi-organ injury induced by IR.

### Epigenetic modification

In addition to oxidative stress and inflammation, epigenetic modification is critically involved in IR-induced senescence. Epigenetic modification refers to changes of genome that occur without any alteration in DNA sequence, including histone acetylation [60, 61], DNA methylation [62, 63], miRNA [62], LncRNA [64, 65], and m6A [66] modification. Growing evidence indicated that epigenetic modification shared complex interaction with cell senescence in multi-organ IR injury. For instance, renal IR injury enhanced the amount of histone H3 acetylation, triggered G2/M arrest and cellular senescence [62], as well as p53 acetylation in the premature senescence of renal tubular epithelial cell (TEC) [67]. Castellano et al. indicated that after IR, aberrant methylation in DNA regions, which involved in cell-cycle control, would result in cell-cycle arrest and senescence in TEC [68]. Meanwhile, m6A modification was found to be a novel mechanism in IR-induced cellular senescence and organ dysfunction [69, 70]. For example, activation of m6A methyltransferase METTL3 after MI can lead to cell-cycle arrest of cardiomyocyte [66]. Besides, Liu et al. reported that miR-493 targets STMN-1 to promote hypoxia-induced epithelial cell-cycle arrest in G (2)/M, leading to renal fibrosis [71]. Moreover, LncRNAs, including SNHG6, AK028326, and Malat1, were recently reported to regulate the p53-senescent pathway in IR-induced kidney injury [64]. Taken together, epigenetic regulation is closely related to multi-organ IR injury, which may serve as a novel therapeutic target to ameliorate or prevent IR injury through regulating cellular senescence.

### The p53/p21 pathway and p16/pR pathway

Under the combined action of oxidative stress [72, 73], inflammation [57], and epigenetic modification [68], the p53/p21^CIP1^ pathway and p16/pRb pathway are activated to induce cellular senescence in IR injury [74–77] (Table 1 and Table 2). Specifically, IR induces the persistent DNA damage response (DDR) and triggers cell signaling cascades reaction involved in the cell-cycle arrest process and DNA repair by activating the p53/p21^CIP1^ [22–24] and p16/pRb [73] pathway. As a result, the cyclin-dependent kinases (CDKs) as well as retinoblastoma protein (RB) are inhibited while the checkpoint activity is enhanced, leading to G1/S (or G2/M) cell-cycle arrest.

**Table 1:** Senescent pathways and outcomes in renal ischemia/reperfusion injury.

Organ	Model	Senescence pathway	Senescence outcomes	Ref.
kidney	C57BL/6 mice; Unilateral renal ischemia 30 min and reperfusion 1d, 7d	p53/p21 pathway p16/pRb pathway	Renal cellular senescence ↑ (SA-β-gal ↑, p53 ↑, p16^INK4A^ ↑, p21^CIP1^ ↑) Renal inflammation↑ Renal fibrosis↑	[64]
kidney	C57BL/6 mice; Unilateral renal ischemia 35 min and reperfusion 1d, 3d, 7d, 14d, 28d	p53/p21 pathway p16/pRb pathway	Renal senescence ↑ (p53 ↑, p16^INK4A^ ↑, p21^CIP1^ ↑) Renal fibrosis↑ Renal function↓	[111]
kidney	C57BL/6 mice; Unilateral renal ischemia 35 min and reperfusion 11d	p53/p21 pathway p16/pRb pathway	Renal senescence ↑ (SA-β-gal ↑, p16^INK4A^ ↑, P19^ARF^ ↑) Renal fibrosis↑ Renal mitochondrial injury↑	[18]
kidney	C57BL/6 mice; Unilateral renal ischemia 30 min and reperfusion 3d, 21d	Not mentioned	Renal cellular senescence ↑ (SA-β-gal ↑) Renal inflammation↑ Renal fibrosis↑	[138]
kidney	C57BL/6 mice; Unilateral renal ischemia 15 min and reperfusion 7d, 14d, 35d	p53/p21 pathway	Renal cellular senescence ↑ (p21^CIP1^ ↑) Renal inflammation↑ Renal fibrosis↑	[114]
kidney	Swiss-Webster mice; Bilateral ischemia 30 min and reperfusion 1, 8d	p53/p21 pathway p16/pRb pathway	Renal senescence ↑ (p16 ^INK4A^ ↑) Renal cell apoptosis↑	[74]
kidney	C57BL/6 mice; Unilateral renal ischemia 30 min and reperfusion 1d, 3d, 7d, 14d, 28d	p53/p21 pathway p16/pRb pathway	Renal senescence ↑ (p21^CIP1^ ↑) Renal inflammation↑ Renal apoptosis↑	[141]
kidney	C57BL/6 mice; Bilateral kidney ischemia 25 min and reperfusion 3d, 7d	p53/p21 pathway p16/pRb pathway	Renal senescence ↑ (p53 ↑, p16^INK4A^ ↑, p21^CIP1^ ↑) Renal inflammation↑ Renal injury↑ Renal fibrosis↑	[90]
kidney	***In vivo***: C57BL/6 mice; Unilateral renal ischemia 30 min and reperfusion 7d ***In vitro***: NRK-49F cells; H_2_O_2_ culture 24 h	p16/pRb pathway	***In vivo***: Renal senescence ↑(p16^INK4A^ ↑) Renal fibrosis↑ Renal inflammation↑ ***In vitro***: Renal senescence ↑(p16^INK4A^ ↑) Renal inflammation↑	[89]
kidney	C57BL/6 mice; Bilateral ischemia 45 min and reperfusion 24 h	p53/p21 pathway p16/pRb pathway	Renal senescence ↑(SA-β-gal ↑, p53 ↑, p21^CIP1^ ↑, p16^INK4A^ ↑) Renal function↓ Renal fibrosis↑ Renal inflammation↑	[81]
kidney	C57BL/6 mice; Bilateral ischemia 32 min and reperfusion 7d	p16/pRb pathway	Renal senescence ↑ (p16^INK4A^ ↑, SA-β-gal ↑, klotho ↓ ) Renal function↓ Renal fibrosis↑	[97]
kidney	BALB/c mice; Right nephrectomy, left kidney ischemia 30 min and reperfusion 24 h	p53/p21 pathway p16/pRb pathway p53 acetylation↑	Renal senescence ↑ (p53 acetylation ↑, p21 ^CIP1^ ↑) Renal apoptosis↑ Renal function↓	[142]

Abbreviations: *NRK-49F cells* normal rat kidney–49 F cells, *LAD* left anterior descending artery, *LCA* left coronary artery, *TAC* Transverse aortic constriction, *hiPSC-MSCs* MSCs derived from human induced pluripotent stem cells.

**Table 2:** Senescent pathways and outcomes in cardiac, hepatic, and brain ischemia/reperfusion injury.

Organ	Model	Senescence pathway	Senescence outcomes	Ref.
Heart	C57BL/6J mice; LAD ischemia 60 min and reperfusion 24 h, 72 h, 1w, 4w	p16/pRb pathway P53/p21 pathway	Cardiac senescence ↑ (SA-β-gal ↑, SASP, p16^INK4A^ ↑, p21^CIP1^ ↑) Cardiac function↓	[56]
Heart	C57BL/6J mice; Coronary artery ischemia 1d, 2d, 7d, 28d	p53/p21 pathway p16/pRb pathway	Cardiac senescence ↑ (SASP ↑, p53 ↑, p21 ^CIP1^ ↑, p16 ^INK4A^ ↑)	[72]
Heart	In vivo: C57BL/6J mice; LCA ischemia 45 min and reperfusion 24 h In vitro: Neonatal rat cardiomyocytes; Hypoxia 12 h and reoxygenation 24 h	p53/p21 pathway p16/pRb pathway	In vivo: Cardiac senescence ↑ (SA-β-gal ↑, SASP ↑, p16 ^INK4A^ ↑, p53 ↑, p19 ↑) Cardiac function↓ In vitro: Cardiac senescence ↑ (SA-β-gal ↑, SASP ↑, p16 ^INK4A^ ↑, p53 ↑, p19 ↑)	[123]
Heart	In vivo: C57BL/6 mice; LAD ischemia 7d In vitro: Primary mice cardiomyocytes; H_2_O_2_ culture 24 h	p53/p21 pathway p16/pRb pathway	In vivo: Cardiac senescence ↑ (SASP ↑, p53 ↑, p16 ^INK4A^ ↑) Cardiac function↓ In vitro: Cardiac senescence ↑ (SA-β-gal ↑, p53 ↑, p16 ^INK4A^ ↑)	[143]
Heart	In vivo: mice and rats; LAD ischemia 1w, 4w; In vitro: neonatal rat cardiomyocytes; hypoxia 16 h and reoxygenation 10 h	p53/p21 pathway	In vivo: Cardiac senescence ↑ (p53 ↑, SA-β-gal ↑) Cardiac function↓ Cardiac fibrosis↑ In vitro: Cardiac senescence ↑ (SA-β-gal ↑, p53 ↑)	[43]
Heart	C57BL/6 mice; LAD ischemia 1d, 1w, 2w, 4w	p53/p21 pathway p16/pRb pathway	Cardiac senescence ↑ (SA-β-gal ↑, SASP ↑, p16 ^INK4A^ ↑, p53↑ and p21^CIP1^ ↑)	[98]
Heart	C57BL/6N mice; TAC 2w, 6 w	p53/p21 pathway p16/pRb pathway	Cardiac senescence ↑ (SA-β-gal ↑, p16 ^INK4A^ ↑, p21^CIP1^ ↑)	[144]
Liver	In vivo: C57/B6 mice; Partial hepatectomy, ischemia 1 h and reperfusion 6 h, 1d, 3d, 5d In vitro: hiPSC-MSCs cell line H_2_O_2_ culture 2 h and normal medium culture 48 h	p16/pRb pathway	In vivo: Hepatic senescence ↑ (SA-β-gal ↑, p16 ^INK4A^ ↑) Hepatic function↓ In vitro: Hepatic senescence ↑ (SA-β-gal ↑, p16 ^INK4A^ ↑)	[57]
Brain	Adult male Wistar rats tMCAO ischemia 1 h and reperfusion 24 h, 3 and 7 d	p53/p21 pathway p16/pRb pathway	In vitro: Cerebral senescence ↑ (lipofuscin granules ↑, SASP ↑, p16 ^INK4A^ ↑, p53↑ and p21^CIP1^ ↑)	[99]
Brain	In vivo: Male Sprague–Dawley rats; left MCAO ischemia 1 h and reperfusion 4d In vitro: Rat brain cortex astrocytes Oxygen-Glucose Deprivation 4 h and Reoxygenation 20 h	p16/pRb pathway	In vivo: Cerebral senescence ↑ (SASP ↑, p16 ^INK4A^ ↑) Inflammation ↑ (NOS2 ↑, MPO ↑) neurological functions↓ In vitro: Cerebral senescence ↑ (SA-β-gal ↑)	[102]
Brain	In vivo: CD1 male mices; tMCAO ischemia 1 h and reperfusion 30 min and 72 h	p53/p21 pathway p16/pRb pathway	Cerebral senescence ↑ (p16 ↑, p21 ↑) Inflammation ↑ (TNFɑ↑, IL6 ↑, Cxcl1 ↑)	[100]

Abbreviations: *NRK-49F cells* normal rat kidney-49F cells, *LAD* left anterior descending artery, *LCA* left coronary artery, *TAC* transverse aortic constriction, *hiPSC-MSCs* MSCs derived from human induced pluripotent stem cells, *OGD/R* oxygen-glucose deprivation/reoxygenation, *tMCAO* transient middle cerebral artery occlusion, *MCAO* middle cerebral artery occlusion.

For instance, oxidative stress happened in IR injury was reported to activate p53-dependent accumulation of p21^CIP1^ and mediate cardiomyocyte senescence, contributing to cardiac dysfunction as well as pathological remodeling [72]. Meanwhile, Qi et al. found that IR-induced inflammation would consequently cause p16^INK4A^ activation and lead to hepatic cellular senescence [57]. Moreover, a recent study confirmed that IR-induced aberrant methylation involved in cell-cycle control and DNA damage would finally result in p53 upregulation and cell-cycle arrest in specific regions [68].

## Cellular senescence is the core mechanism of transition from acute to chronic stage after IR

**Fig. 4:**
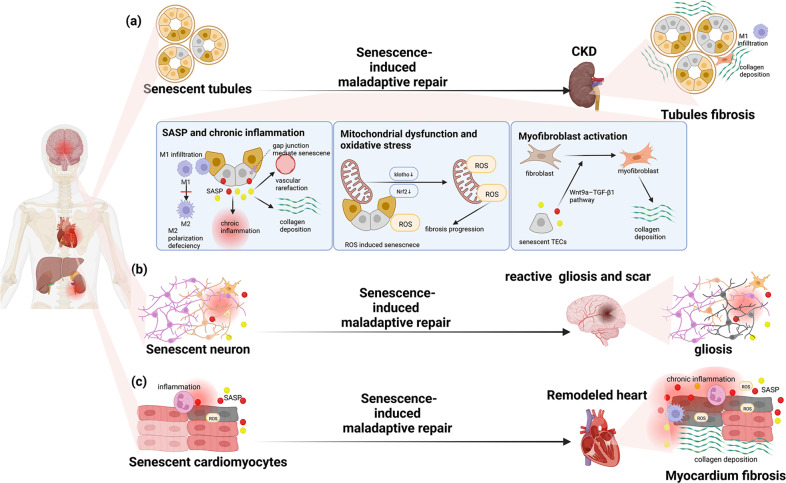
Mechanisms of senescence-induced acute injury to chronic stage transition. **a** After kidney IR injury, IR-induced cellular senescence is a major initiative of AKI-to-CKD transition, which is mediated by SASP and chronic inflammation, mitochondrial dysfunction and oxidative stress and myofibroblast activation. Firstly, the existence of senescent TECs will cause persistent inflammation and lead to M1 infiltration and M2 polarization deficiency. Besides, Senescence burden in tubule is aggravated via gap junction and further contributes to chronic inflammation, leading to collagen deposition and vascular rarefaction. Secondly, Mitochondrial dysfunction and ROS generation caused by cellular senescence may result in renal fibrosis. Finally, fibroblast will be activated via Wnt9a-TGF-β1 pathway and intensify renal fibrosis. **b** After ischemic stroke, IR-induced senescent neurons may lead to reactive gliosis and scar forming. **c** After heart IR injury, IR-induced senescent cardiomyocytes result in heart remodeling through inflammation and SASP.

Besides the acute organ dysfunction, cellular senescence was recently confirmed to make a contribution to the transition from acute to chronic stage in different organs after IR. Here, we will combine the recent research progress and discuss the pivotal role of cellular senescence in acute kidney injury to chronic kidney disease (AKI-to-CKD) transition, cardiac injury progression and in ischemic stroke-induced glial scar and cerebral fibrosis (Fig. 4).

### Cellular senescence in AKI-to-CKD transition

The kidney receives 20% of cardiac output and consumes 10% of body oxygen, which makes it vulnerable to IR injury [78]. Moreover, although patients suffer from mild AKI can restore normal renal function, more than 70% of them experience renal maladaptive repair, and more than 50% of them will gradually develop into CKD [79], becoming the fifth leading cause of death by 2040 [78, 80]. Hence, research on the underlying mechanism of AKI-to-CKD is becoming increasingly attractive, and one of the recent snapshots is the senescent TECs and senescence-associated fibrosis [9, 74, 81–83]. To be specific, cellular senescence participates in AKI-to-CKD through multiple mechanisms, such as SASP, chronic inflammation, mitochondrial dysfunction, oxidative stress, and myofibroblasts activation.

#### SASP and chronic inflammation

On the one hand, senescent TECs caused by renal IR remain metabolically active and adopt SASP to release inflammatory cytokines and other fibrotic factors that serve as contributors to risk factors in maladaptive repair [84] and renal fibrosis [85, 86], such as collagen deposition, vascular rarefaction and chronic inflammation [86–89]. Interestingly, recent evidence found that senescent TECs could interrupt the macrophage polarization, increasing M1 infiltration and impaired M2 polarization to induce chronic inflammation in kidney [90]. On the other hand, senescent TECs caused by renal IR might further induce DNA damage response in neighboring cells by cell-cell contact via the gap junction and cause cellular senescence in intact bystander TECs and fibroblasts [91, 92], which might enhance SASP release and lead to renal maladaptive repair and senescence-associated fibrosis [81].

#### Mitochondrial dysfunction and oxidative stress

On the one hand, Klotho deficiency in IR-induced senescent TECs would further promote mitochondrial injury, ROS generation, and fibrotic lesions [18, 93]. Meanwhile, senescent TECs would downregulate Nrf2 and attenuate anti-oxidative response [39, 88, 94, 95], leading to AKI-CKD transition [35]. On the other hand, senescent cells produce and secrete ROS to induce further DNA damage response in neighboring TECs via gap junction-mediated between adjacent cells [92]. These pieces of evidence suggest that oxidative stress and mitochondrial dysfunction are involved in senescence-induced chronic renal injury.

#### Myofibroblasts activation

Another key characteristic of maladaptive repair is the activation of numerous myofibroblasts which make contribution to the deposition of collagen and other pro-fibrotic components in kidney [86]. Recently, increasing evidence showed that senescent TECs would enhance myofibroblasts activation via SASP generation in an epithelial-mesenchymal transition (EMT) manner [96]. Meanwhile, senescent TECs would generate TGF-β1, a factor contribute to interstitial fibroblast proliferation and transition to myofibroblasts [97]. Furthermore, a reciprocal activate loop between senescent TECs and myofibroblasts was mediated by Wnt9a-TGF-β1 pathway, which promoted and accelerated the pathogenesis of renal fibrosis [97]. Taken these pieces of evidence together, myofibroblasts activation is substantially relevant to senescence TECs after renal IR.

### Cellular senescence in cardiac IR-induced heart remodeling

Consistent with the kidneys, hearts are also prone to suffer from IR injury since they are organs with high energy demand. As a process of cardiac repairment after IR, cardiac remodeling is initially adaptive to the damage induced by IR in the short term, but becomes maladaptive due to the sustained stress, resulting in progressive and irreversible cardiac dysfunction and heart failure [72]. Among all the culprits, cellular senescence induced by IR injury plays an essential role in heart remodeling and transition into chronic heart injury [56, 72].

Recent studies showed that senescent cardiomyocytes induced by cardiac IR would secret SASP [98] and further activate the maladaptive cardiac remodeling, including cellular hypertrophy, inflammation, fibrosis, and attenuation of regeneration, which ultimately contribute to cardiac chronic fibrosis [56, 98]. At the same time, studies revealed that IR-induced senescent cardiomyocyte might provide cardioprotective effects by promoting cellular senescence of fibroblast, which promoted neonatal heart regeneration by accelerating cardiomyocyte proliferation and inhibiting cardiac fibrosis [98]. Together, these results present pieces of evidence of involvement of senescent cells in the acute to the chronic transition of cardiac dysfunction after IR.

### Cellular senescence in ischemic stroke-induced glial scar and cerebral fibrosis

In addition to the kidney and heart, brain is also prone to suffer from I/R injury. A recent study also showed that neuron senescence served as a pathogenic mechanism for ischemic stroke-induced brain damage [99, 100], which might result in glial scar and cerebral fibrosis [101].

On the one hand, senescent neurons induced by stroke have a significant increase in the expression of SASP including IL6, TNFα, and CXCL1 [99, 100, 102], which induce cerebral inflammation microenvironment forming and extracellular matrix(ECM) deposition by a pericyte-dependent manner [101]. On the other hand, in the early stages of cerebral IR injury, glial cells especially astrocytes are activated by pro-inflammatory cytokines generated by senescent neurons [101, 103, 104]. Moreover, reactive astrocytes may secrete a myriad of adhesion molecules and pro-inflammatory cytokines, such as VCAM-1, ICAM-1, IL-1β, IL-6, and TNF-α [105], which could involve in a cyclic process of consecutive activation, consequently resulting in cerebral fibrosis, glial scar [106, 107] and regeneration failure in ischemic zones [108].

## Therapies that target cellular senescence after IR injury

**Fig. 5:**
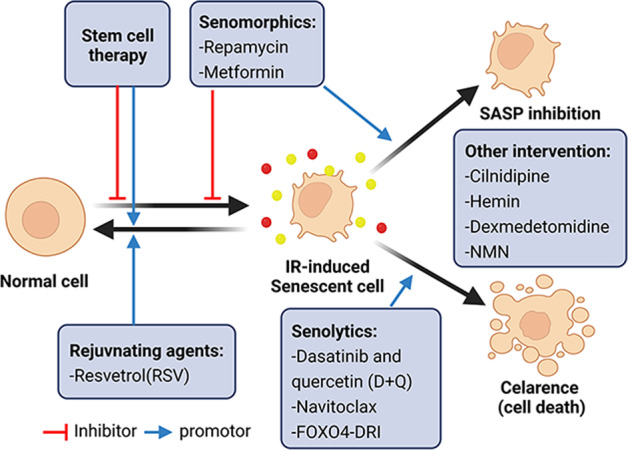
Therapies that target cellular senescence to alleviate IR injury. Several kinds of intervention including senolytics, senomorphics, rejuvenating agents, stem cell therapy, and other intervention, are developed to attenuate the deterioration brought by senescent cells.

As mentioned above, premature cell senescence caused by IR injury plays a pivotal role in multiple organs’ acute and chronic dysfunction. Therefore, treatments targeting cellular senescence means a promising prospect in IR injury. Based on the observation, the elimination of senescent cells is mostly beneficial and seems to have few long-term deleterious consequences, researchers have identified various novel agents and strategies to achieve this, which were also called ‘senotherapeutic’ strategies [109]. In summary, it can be classified into four classifications: pharmacological agents named ‘senolytics’ that clear senescent cells, ‘senomorphics’ that prevent harmful effects of senescent cells [110], rejuvenating agents that stimulate SIRT1 to alleviate senescence, and stem cell therapy (Fig. 5).

### Senolytics

Formed by the words “senescence” and “lytic” (destroying), senolytics include pharmacological agents targeting the specific elimination of senescent cells. Increasing pieces of evidence have shown that senolytics might be effective tools to eliminate senescent cells to treat age-related diseases and IR-induced cellular senescence [34]. As for senolytics, the most reported include the combination of dasatinib and quercetin (D + Q), Navitoclax and FOXO4-D-Retro-Inverso peptide (FOXO4-DRI).

A growing body of pieces of evidence suggest that D+Q treatment could disable pro-survival networks and eliminate senescent cells in IR-related organ dysfunctions [111, 112]. For instance, D+Q could reduce senescent cell burden, promote TECs’ proliferation, ameliorate renal fibrosis, and decrease renal inflammation in IR-induced kidney disorders. [111] Moreover, treatment of aged animals with D+Q was reported to eliminate senescent cells and diminish cell-free mitochondrial DNA (cf-mt-DNA) release, attenuating the IR-associated cardiac injury and prolonging the survival of aged cardiac allografts [112]. Meanwhile, D+Q were reported to be beneficial in reducing senescent cell levels and improving renal transplantation outcome [109].

Navitoclax (ABT-263) is a kind of Bcl-2 inhibitors that induces senescent cell apoptosis and death in aged mice [34, 113] and promotes rejuvenation of stem cells for tissue regeneration [109]. A recent study revealed that ABT-263 could reduce senescent cell burdens and restore a regenerative phenotype with improved function, increased tubular proliferation, and reduced fibrosis in the kidneys after renal IR [114]. Furthermore, ABT-263 was shown to eliminate cerebral IR-induced neural cell senescence, reduce the infarct volume and improve neurological function in animal models [102]. Elimination of senescent cells with Navitoclax during cardiac IR injury was proved to be a potential novel therapeutic avenue in improving patients outcomes following cardiac IR [56].

FOXO4-DRI, as a novel cell-penetrating peptide, is designed to interfere with the endogenous p53-FOXO4 interaction [36] and potentially target senescent cells by influencing the p53-dependent apoptosis [36]. Although the clearance of senescent cells with FOXO4-DRI was reported to restore renal function and reduce inflammation markers in the kidney of aged mice [35, 36], whether it is therapeutically feasible in IR injury still needs further exploration. Other approaches rely on immune-system-mediated clearance of senescent cells are emerging consecutively and might become promising methods in mitigating senescent burden after IR-induced senescence in the near future [115].

### Senomorphics

The use of senomorphic agents is an alternative to complete the clearance of senescent cells through senolysis against IR injury. Senomorphics is designed to prevent cells occurring growth arrest as well as to disrupt the generation and secretion of SASP while keep the cells alive. This method could interfere with the pro-inflammatory nature of IR-induced cellular senescence and potentially delay the critical effects of IR injury and organ aging [110].

The most commonly reported senomorphics are rapamycin and metformin. Rapamycin is a kind of mTOR inhibitors that have been reported to regulate autophagy and inhibit cellular senescence in renal IR injury [81, 116, 117] via enhancing Wnt signaling [110]. Metformin, an AMPK activator, has been reported to attenuate IR-induced mitochondrial dysfunction [118], decrease the level of p16^INK4a^ and p21^CIP1^ and inhibit the release of SASP-related cytokines [119]. Increasing evidence also showed that Lipoxin A4 might stimulate inflammation resolution and inhibit cellular senescence in septic AKI [46].

### Rejuvenating agents

Rejuvenating agents specifically refer to the interventions that stimulate SIRT1 to alleviate senescence. In mammals, SIRT1 is well-characterized to enhance cell proliferation and inhibit cellular senescence through the suppression and deacetylation of p53, [120–122]. Resveratrol (RSV), as the most reported agonist of SIRT1, is potentially to protect organs against IR-induced premature cellular senescence [123]. Further clinical studies are needed to confirm and elaborate the protective effects on the application of RSV in IR injury.

### Stem cell therapy

Stem cell therapy is another promising treatment for IR-induced senescence. Multipotent mesenchymal stem cells (MSCs), being a category of adult stem cells springing from the mesoderm, with multi-directional differentiation and self-renewal potential, have recently emerged as a key player in regenerative medicine and clinical translational research [124–126]. Typically, MSCs have been extensively studied to inhibit premature senescence by protecting against the IR-induced pro-oxidative state, cell-cycle inhibition [93], and chronic fibrosis [127].

Recently, the increasing underlying mechanism of MSCs against IR-induced senescence has been clarified. On the one hand, MSCs can exert immunomodulatory ability via secreting soluble factors or direct contact with the immune cells, and transform them into an anti‐inflammatory phenotype and further inhibit cellular senescence [128]. On the other hand, MSCs can secret extracellular vesicles (EVs) to inhibit the generation of SASP in senescent cells [129, 130]. For instance, cell-cycle arrest of myocardiocytes after MI can be alleviated by MSC-EVs carrying miR-150-5p via downregulation of TXNIP [131, 132], or by MSC-EVs targeting miR-497/Smad7/TGF-β pathway [133]. Yu et al. also found that EVs carrying mi-202-3p could protect neurons from IR injury via downregulating TLR4-mediated inflammation response [124]. Xiao et al. point that MSC-EVs reduce endothelial cell senescent burden and activate angiogenesis through miR-146a/Src pathway [134].

With the further study of MSCs, more and more novel treatments derived from MSCs have been contrived. For example, prior clearance of senescent cells enhanced the beneficial effects of KIM‐MSC on cellular senescence [135]. Yu et al. suggested that mi-R217 inhibitor may enhance MSCs’ repair of vascular damage and senescence via SIRT1 upregulation [136]. Klotho gene-modified MSCs were recently found to inhibit cellular senescence and show elevated anti-fibrotic effects in kidneys after IR [137]. In general, stem cell therapy provides an innovative approach in IR injury treatment, but the mechanism and clinical application still need further study.

### Other interventions

Several other interventions were also found effectively targeting cellular senescence in IR injury. For instance, it was reported that Cilnidipine could prevent hypoxia-induced mitochondrial hyperfission and myocardial senescence [43]. Dexmedetomidine, a highly selective α2 adrenergic receptor (α2-AR) agonist, was proved to be useful in inhibiting cellular senescence and IR-induced renal fibrosis [81]. Moreover, Nicotinamide mononucleotide (NMN) could attenuate renal interstitial fibrosis by suppressing DNA damage and senescence of TECs in AKI [138]. More and more further study may reveal the potential clinical application value of such interventions.

## Conclusion and outlook

Increasing pieces of evidence are revealing new insight into the crucial role of cellular senescence in IR injury. Up to now, IR-induced mochondrial dysfunction and oxidative stress, inflammation, epigenetic modification, and activation of p53/p21 and p16/pRb pathways have been reported to ultimately cause cellular senescence. At the same time, IR-induced cellular senescence contributes to the transition from acute organ injury to chronic dysfunction through inflammation, oxidative stress, mitochondrial dysfunction, and myofibroblast activation. However, the currently known function of cellular senescence in IR injury is just the tip of iceberg. For instance, senescence in different types of cells would bring different outcomes. As is mentioned in hepatic and cardiac IR injury, the senescence of hepatic stellate cells [139] and cardiac fibroblast [98] played a protective role in the repair process. Thus, advanced technology such as organoid model [140] and single-cell sequencing should be adopted to explore the precise role of cellular senescence in acute organ injury to chronic disease transition.

With the growing awareness of the importance of cellular senescence in IR-induced acute organ injury and chronic dysfunction comes the potential to target cellular senescence with novel therapeutic strategies. Senolytics, senomorphics, SIRT1 agnoist and stem cell therapy are the most well-reported and promising treatments for cellular senescence in in vivo and in vitro experiments. However, in light of the fact that cellular senescence is instrumental in preventing dangerous DNA mutations, it is important to assess carefully the effects and safety of these drugs to attenuate IR injury in humans.

In a word, more and more researches will certainly shed light on the role of cellular senescence in IR injury and acute to chronic dysfunction transitions. This pervasive disease will certainly be overcome with further research and the novel therapies deserve the higher priority.

## Data Availability

The data used to support the findings of this study are available from the corresponding author upon request.
